# Multiscale approach predictions for biological outcomes in ion-beam cancer therapy

**DOI:** 10.1038/srep27654

**Published:** 2016-06-14

**Authors:** Alexey Verkhovtsev, Eugene Surdutovich, Andrey V. Solov’yov

**Affiliations:** 1 MBN Research Center, Altenhöferallee 3, 60438 Frankfurt am Main, Germany; 2 Instituto de Física Fundamental, CSIC, Serrano 113-bis, 28006 Madrid, Spain; 3 Department of Physics, Oakland University, Rochester, Michigan 48309, USA

## Abstract

Ion-beam therapy provides advances in cancer treatment, offering the possibility of excellent dose localization and thus maximising cell-killing within the tumour. The full potential of such therapy can only be realised if the fundamental mechanisms leading to lethal cell damage under ion irradiation are well understood. The key question is whether it is possible to quantitatively predict macroscopic biological effects caused by ion radiation on the basis of physical and chemical effects related to the ion-medium interactions on a nanometre scale. We demonstrate that the phenomenon-based MultiScale Approach to the assessment of radiation damage with ions gives a positive answer to this question. We apply this approach to numerous experiments where survival curves were obtained for different cell lines and conditions. Contrary to other, in essence empirical methods for evaluation of macroscopic effects of ionising radiation, the MultiScale Approach predicts the biodamage based on the physical effects related to ionisation of the medium, transport of secondary particles, chemical interactions, thermo-mechanical pathways of biodamage, and heuristic biological criteria for cell survival. We anticipate this method to give great impetus to the practical improvement of ion-beam cancer therapy and the development of more efficient treatment protocols.

The damaging effects of ionising radiation have been studied for many years. Regardless whether the goal is to kill tumourous cells using radiotherapy or to protect cells exposed to radiation, the relation of physical conditions to the biological effects has always been the major challenge for radiobiology^1^. Historically, this relation is presented as the dependence of the clonogenic cell survival probability on the deposited dose. These dependencies are known as survival curves and they are the starting point for any treatment plan. Having an objective of inactivating certain fraction of cells in a given region, radiation oncologists determine the dose that has to be delivered in this region. Photons have been the most common radiation used for therapy and a vast experience has been gained for different cells in different conditions. The biological diversity of survival curves is such that there was no hope to predict their shape theoretically.

When ion-beam modality^2^,^3^ became a reality in 1990s^4^, the survival curves for ions were obtained by and large either experimentally or semi-empirically, based on the photon experience. However, the MultiScale Approach (MSA) to the assessment of radiation damage with ions suggested a possibility to predict the survival curves theoretically based on major physical effects^5^. This possibility arises because in the case of ions the physical and chemical actions may be more definitive for the biological response. The approach considers these effects on different scales in space, time, and energy and explores their relation to biological damage. The realisation of the goal of the MSA was described in the form of a recipe for calculating survival curves^5^. One of the components of this recipe is the criterion of significance of a lesion that inactivates a cell. This criterion required further justification that has been carried out in this paper. We demonstrate capability and predictive power of the above recipe by an extensive comparison with experimental data for numerous mammalian cancer and normal fibroblast cell lines, such as A549, AG1522, A172, CHO, V79, HeLa, NB1RGB, and EAhy926, under different irradiation conditions.

In both x-ray and ion-beam irradiations, secondary electrons, free radicals, and other reactive species do the major damage to the cells, but the qualitative features such as the dose dependence of the probability of cell survival are different for these radiation modalities. When tissue is exposed to x-rays, the dose distribution in the plane perpendicular to the beam axis on the cellular (10 μm) scale is uniform. Therefore, the model for determining the probability of cell survival can be built on a single physical quantity such as dose^1^. With ions, the above picture changes since the radial distribution of dose varies on the nanometre scale and the dose on the cellular and sub-cellular scales becomes a probabilistic function of a number of ions traversing a given target^5^. Physical parameters, such as number density of reacting species or their fluence, describe radiation damage in a more straightforward way. This is why all semi-empirical approaches^6^,^7^ (incorporated in existing treatment protocols) based on the linear-quadratic (LQ) model,


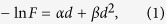


where *F* is a surviving fraction of cells, *d* is the dose, with the coefficients α and *β* being determined empirically, become artificial. However, the full potential of ion-beam therapy can only be realised if the fundamental mechanisms^8^,^9^ leading to lethal damage of cells under ion irradiation are well understood. This understanding is fostered by means of the MSA. Having been developed specifically for ions, this approach joins the knowledge about ion stopping in the medium, the production of secondary electrons and other reactive species in the vicinity of ion’s path, the transport of these species, the interaction of secondary particles with biomolecules, relaxation of energy in the medium that leads to thermo-mechanical damage due to the formation of nanoscale shock waves^10^,^11^, and the interaction of secondary species with DNA molecules to calculate the probability of important lesions, such as double and single strand breaks (DSBs and SSBs) per unit length of ion’s path^5^. On this basis, a criterium for lethality of damage is established, and, finally, the probability of production of lethal lesions is obtained. All these features make the MSA unique because it can predict the macroscopic effects of ionising radiation based on the inclusive scenario and fundamental science. The criterium for lethality of damage suggested in ref. 5 is based on the idea that among different DNA lesions caused by interaction with reactive species the multiply damaged sites with a sufficient complexity may not be repaired^12^,^13^. In the formulated recipe for the assessment of biodamage^5^, it was postulated that a lesion combined of a DSB and at least two other simple lesions such as SSB within two DNA twists is lethal. In this paper, we apply and justify this criterion for all cell types.

In regard to irradiation with heavy charged particles, the key assumption adopted in the MSA following refs 12, 14 and 15 is that the leading cause of cell inactivation is the complexity of nuclear DNA damage. However, this may change under different conditions, e.g., when biodamage takes place in presence of sensitising nanoparticles^16^,^17^. Indeed, it is currently established that radiosensitising nanoparticles delivered to the cells are preferentially localised outside cell nuclei^18^. Therefore, the damage of other organelles may become more important.

## Results

**Figure 1:**
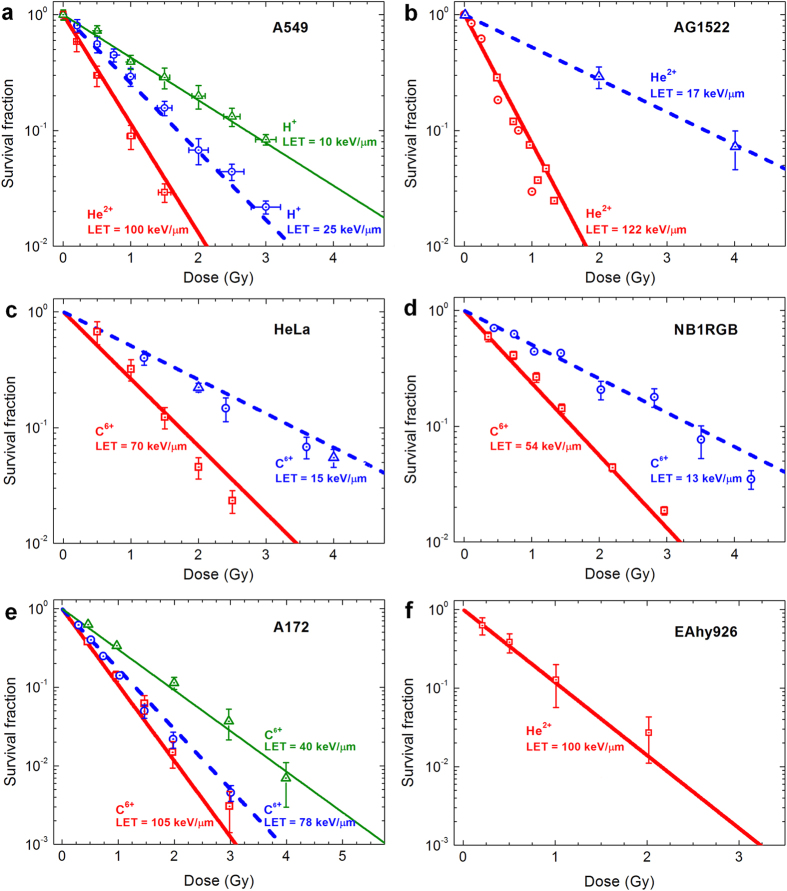
Survival curves for different human cell lines: adenocarcinomic A549 cells (**a**), normal fibroblasts AG1522 (**b**), cervical cancer HeLa cells (**c**), normal skin fibroblasts NB1RGB (**d**), glioblastoma A172 cell line (**e**), and endothelial EAhy926 cells (**f**). The calculated survival probabilities are shown with lines and experimental data from refs 51, 52 (A549), refs 23, 53 and 54 (AG1522), refs 55, 56 (HeLa), refs 48, 49 (NB1RGB), refs 49, 57 (A172), and ref. 58 (EAhy926) are shown by symbols.

Figure 1(a,b) shows the survival curves for human adenocarcinomic A549 cells and normal fibroblasts AG1522, irradiated with protons and alpha-particles at different values of linear energy transfer (LET), which is approximately equal to the stopping power, d*E*/d*x*, with *E* being the ion’s energy and *x* the longitudinal coordinate. The calculated curves (lines) are compared to the experimental data (symbols) on survival of the same cells in the same conditions.

The probability of cell survival, Π_surv_, decreases exponentially with respect to the yield of clustered damage events, which are deemed to be lethal for cells (see section *Methods* for a detailed explanation),


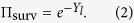


This yield, *Y*_*l*_, linearly increases with dose if the probability of at least one ion to traverse a cell nucleus is sufficiently large (see Eqs (13–15) in Methods). This results in an exponential dependence of cell survival on dose, which is a common feature for cells irradiated with ions^5^. Different cell lines have different cross sectional area of their nuclei, and, thus, the average distance 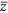 of the ion’s traverse through the nucleus (see Table 1). This results in different slopes of the survival curves calculated for A549 and AG1522 cell lines at comparable values of LET. More comparisons of calculated survival curves for other human cell lines with experiments are presented in Fig. 1(c–f).

**Figure 2:**
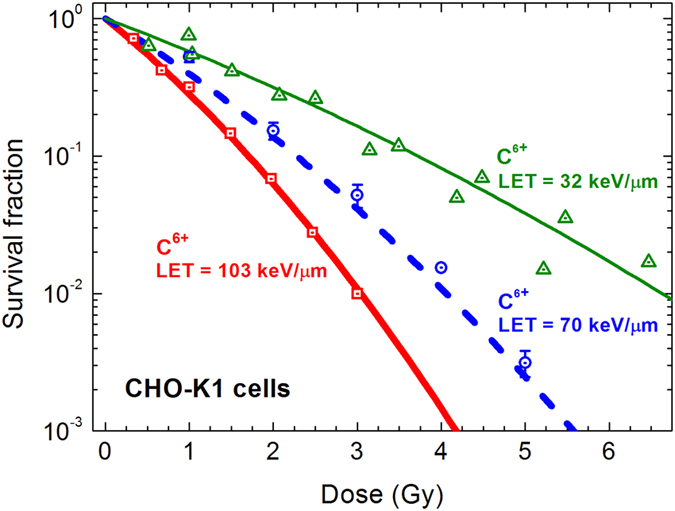
Survival curves for a repair-efficient CHO cell line. The calculated survival probabilities are shown with lines and experimental data from refs 20, 21 are shown by symbols. The survival curves are calculated using Eq. (5) with the probability (4), where *χ*_0_ = 0.35 and *χ*_1_ = 0.04.

In some cases, there is an evidence that survival curves as a result of irradiation with ions can be “shouldered” consequent to successful damage repair; i.e., in the language of LQ model, Eq. (1), the coefficient *β* may be noticeably large^19^. In the framework of the MSA, this means that in these cases, some complex lesions, deemed to be lethal, can still be repaired. A possibility of repair leads to the deviation from a purely exponential behaviour of survival probability and the appearance of shoulders in survival curves, which have been observed in experiments for specific cells^20^,^21^. An example for such curves for the repair-efficient Chinese hamster CHO cell line is shown in Fig. 2. Below, in the *Discussion* section, we explain the MSA formalism accounting for such a damage repair.

**Figure 3:**
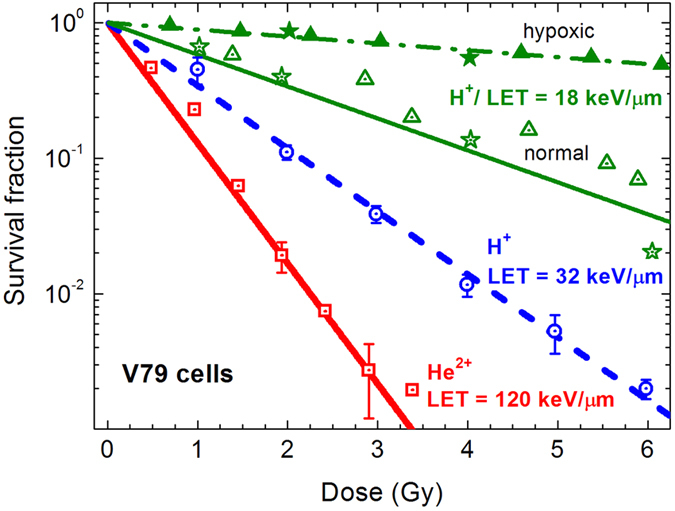
Survival curves for Chinese hamster V79 cell line. The calculated survival probabilities are shown by lines and experimental data from refs 23, 24, 25, 26 are shown by symbols. Experiments performed under normal and hypoxic conditions are depicted by open and closed symbols, respectively.

For a more complete picture, we analysed the widely studied Chinese hamster V79 cells irradiated with protons and alpha-particles (see Fig. 3), thus confirming the capability of the MSA to reproduce a large number of experimental results, based on the understanding of fundamental molecular and nanoscale mechanisms of radiation damage. With this understanding, it becomes possible to evaluate the probability of cell survival under different environmental conditions of irradiated targets. This issue is crucial for medical applications because in many clinical cases, especially in the centre of large tumours, one can find regions with reduced oxygen concentration^22^. It is established that the presence of molecular oxygen substantially changes chemical interactions with biological molecules as it affects both the content of reactive species and the possibility of damage fixation. The survival curves calculated for the V79 cells irradiated under aerobic and hypoxic conditions are presented in Fig. 3 alongside with the corresponding experimental data^23^,^24^,^25^,^26^. Under hypoxic conditions, the experimental studies (closed symbols) were performed at high level of hypoxia, since they were carried out in the atmosphere of nitrogen with no addition of oxygen.

**Figure 4:**
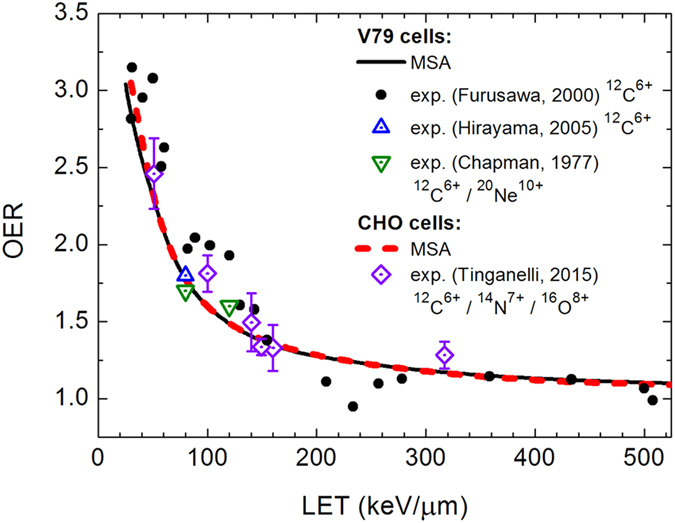
Oxygen enhancement ratio at the 10% survival level for V79 and CHO cells irradiated with carbon ions. Symbols denote the experimental data taken from refs 22, 27, 28, 29.

Evaluation of cell survival under different environmental conditions allows one to analyse the oxygen enhancement ratio (OER). It is defined as the ratio of the dose delivered under hypoxic conditions to that under normal aerobic conditions, leading to the same biological effect, such as the probability of an irradiated cell inactivation^1^. The OER is about 3 for low-LET radiation and gradually approaches unity as the LET of the radiation increases. In Fig. 4, we present the OER at the 10% survival level calculated for CHO and V79 cells irradiated with carbon ions. The calculated curves cover a broad range of LET and are compared to existing experimental results for carbon and heavier ions. The MSA adequately describes the main features of the OER as a function of LET: namely, it predicts the decrease of the OER with increasing the LET and its asymptotical value equal to unity at high LET. It also provides good quantitative agreement with experimental data^27^ in a broad range of LET. At the LET ranging from approximately 100 to 150 keV/*μ*m, where the relative biological effectiveness (RBE) for carbon ion beams reaches its maximal value^27^, the OER is within the range from 1.5 to 2.0 and nicely agrees with different experimental measurements^22^,^28^,^29^.

## Discussion

The effect of reacting species formed near ion paths strongly depends on their transport. If the latter were driven exclusively by diffusion, free radicals would not be able to propagate further than a few nanometres from the ion’s path. Their high reactivity in the region of their high concentration would result in their annihilation^30^. Shock waves predicted in ref. 10 significantly change this picture as they are capable of effectively propagating the reactive species to much larger distances. For instance, a shock wave produced by a single carbon ion at the Bragg peak (LET ≈900 keV/*μ*m) propagates free radicals to the distances of about 10 nm from the ion’s track^10^, and this value gradually decreases with decreasing projectile’s velocity and charge. In the plateau region of the depth-dose distribution (LET ~10–20 keV/*μ*m), the shock wave is much weaker, if at all significant, and the reactive species may produce damage to the DNA in a narrower region around the ion’s path^30^. The low-LET (less than 20 keV/*μ*m) survival curves, shown in Figs 1 and 3 were calculated with an effective distance of free radicals distribution equal to 5 nm. This value corresponds to a characteristic diffusion range for radicals in mammalian cells. Beyond this distance, the probability of DNA damage induced by OH^·^ radicals rapidly decreases^31^,^32^.

The probability for lesion production by free radicals is also sensitive to environmental conditions of irradiated targets. At the early stages of the radiation-matter interaction, a decrease of the concentration of diluted oxygen in the cell environment can modify the water radiolysis process that results in modification of primary DNA damage yields^33^. On the other hand, it has been discussed that the effect of oxygen can be explained mainly by chemical repair or oxygen fixation of primary DNA damages, which come into play at later stages of the radiation-matter interaction depending on the oxygen concentration^32^,^33^. In the case of hypoxic conditions, the damage induced by secondary species may be repaired chemically through reduction of DNA radicals by endogenous thiols such as glutathione or other sulfur-containing cellular constituents^34^, thus decreasing the number of individual and clustered DNA lesions processed by enzymatic repair mechanisms. All these mechanisms suggest that under hypoxic conditions, the average probability for radical-induced lesion production at a given distance from the ion’s path should be smaller than that in the aerobic environment. Experimental survival probabilities of cells irradiated under hypoxic conditions (Fig. 3) are nicely described with the probability, which is two times smaller than that used to describe aerobic conditions (see section *Methods*); this corresponds to experimental data on the induction of DSBs and non-DSB clustered DNA lesions in mammalian cells at normal concentration of oxygen and at deep hypoxia^32^. Reduction of the oxygen concentration under hypoxia results in a decrease in the rate of formation of free radicals and, thus, in a decrease in the effectiveness of free radicals to produce DNA damage.

In the case of irradiation with ions, Π_surv_ reveals by and large an exponential behaviour on dose and the survival curves are straight lines in a semi-logarithmic plot, see Fig. 1. As noted above, the deviation from a purely exponential behaviour of survival probability can be explained by a possibility of repair of complex lesions. In these cases, a biological parameter, the probability of a successful repair of a complex lesion, *χ*, is introduced and Eq. (2) transforms into


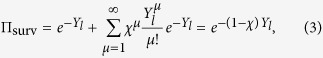


where each term in the sum represents the probability of exactly *μ* complex lesions to be induced multiplied by *χ*^*μ*^, since all of these lesions must be repaired.

The probability of repair of a complex damage may depend on the cell’s response to radiation, which involves specific biological mechanisms of damage repair^35^. Although the exact form of this dependence is unknown, the simplest function of probability, *χ*, can be introduced as a linear function of *Y*_*l*_,


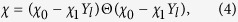


where the positive parameters *χ*_0_ and *χ*_1_ of the function of probability are likely to depend on a cell line, cell phase, and irradiation conditions, and Θ(*x*) is the Heaviside step function. A study of these dependencies as well as biological reasons for such a functional dependence requires a significant effort and goes beyond the scope of the present paper. The probability *χ* gradually approaches zero with increasing the number of lesions until it becomes equal to zero at a critical value, 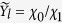, which depends, in particular, on dose and LET (see *Methods*).

When the probability of repair of complex lesions is taken into account, the survival probability transforms into


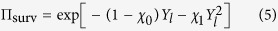


below the critical value 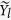 and into Eq. (2) above it. These equations explain the meaning of the critical value 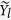 as the transition point in the survival curve from the linear-quadratic to the linear regime. Such a behaviour can be observed in the experimental curves presented in Figs 1 and 2. Equations (2 and 5) can further be expressed as a function of the system parameters (see Equations (18 and 20) in *Methods*), thus providing the molecular-level justification of the empirical LQ model.

The survival curves for CHO cells (Fig. 2), which describe irradiation with carbon ions with LET = 32, 70 and 103 keV/*μ*m, were obtained with the values *χ*_0_ = 0.35 and *χ*_1_ = 0.04. Depending on the value of LET, the maximal dose at which repair of complex lesions is still possible ranges between about 5 Gy (for LET = 103 keV/*μ*m) and 11 Gy (for LET = 32 keV/*μ*m). At higher doses, the probability of repair, *χ*, is equal to zero and the survival curves become purely exponential functions of dose. Survival probabilities for different human cell lines, presented in Fig. 1, are calculated with *χ* = 0, i.e., these probabilities decrease exponentially even at small doses. Even though some of these survival curves (e.g., Fig. 1(c)) can be improved by introducing *χ*, the further analysis of these dependencies goes beyond the scope of this work. Here, we simply demonstrate that rather good agreement with experimental data can be achieved in many cases without accounting for the damage repair and thus the associated empirical inputs.

## Conclusions and Outlook

In conclusion, novel techniques of radiation therapy, such as ion-beam therapy, can be fully exploited only after the complete scenario of biological damage consequent to irradiation with ions is well understood. This understanding is fostered by means of the MultiScale Approach to the physics of radiation damage with ions – an analytic approach that constructs the scenario of biodamage accounting for the key physical, chemical, and biological effects that take place on different spatial, time, and energy scales. Our extensive comparison with experimental data on survival probability of a broad range of cell lines, irradiated with protons and heavier ions at different values of linear energy transfer and under aerobic and hypoxic conditions, demonstrates the capability of this method to accurately predict the probability of cell survival and related phenomena such as oxygen enhancement ratio. The advantages of the method allow one to extend it to many other cell lines, including radiosensitive and radioresistive cells, different cell phases, irradiation conditions (e.g. in the presence of sensitisers) and make predictive evaluation of radiobiological effects. This analysis will be continued as the predictions are experimentally verified in the future. Then, judgements on practical implementation of the new methodology in treatment planning can be made. Finally, we want to emphasise that the understanding of the phenomena at play on a solid physical basis is crucial for technological advances of new treatment techniques.

## Methods

The main aspects addressed by the MSA are ion stopping in the medium, the production and transport of secondary electrons and free radicals produced as a result of ionization and excitation of the medium, the interaction of secondary particles with biomolecules, the analysis of induced damage, and the evaluation of the probabilities of subsequent cell survival. A comprehensive description of different aspects of the MSA is given in ref. 5. In this section, we briefly outline the formalism used for the estimation of radiobiological effects within this approach.

The calculation of a survival curve starts with establishing the relation between physical effects and the lethality of radiation damage. In this work, we have focused our attention on cell damage brought about by pathways that involve only nuclear DNA damage. It was assumed that a complex lesion combined of a DSB and at least two other simple lesions within two DNA twists is lethal for a cell^5^. Thus defined criterion is based on the well-established hypothesis^12^,^13^,^15^ that a clustered DNA damage, i.e. a combination of several simple lesions within a certain DNA region, is lethal. We kept this criterion for all cell types.

The multiple damage sites containing clustered damage are brought about by several independent agents, such as secondary electrons, free radicals, or other reactive species^36^,^37^. In this analysis, each simple lesion requires a separate agent attacking a DNA segment. The average number of lesions produced at a distance *r* from the ion’s path is defined as:


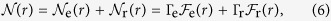


where the functions 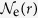 and 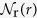 define the average number of lesions like SSBs, base damages, etc., done by secondary electrons and other reactive species (free radicals, pre-solvated and solvated electrons), respectively.

The calculation of the probability for the clustered damage to occur starts with the calculation of the number of secondary electrons incident on a given DNA segment^36^. This number is averaged over all angles and as a result the number of hits with secondary electrons, 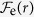, is obtained as a function of distance from the ion’s path. The function 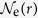, is then calculated by multiplying 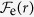 by the probability of inducing a lesion per hit, Γ_e_. The same is done for free radicals and other reactive species. Function 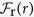 includes the physics pertinent to transport of reactive species, such as the relaxation of ionisation energy in the medium and the (predicted) cylindrical shock wave around the ion’s path^5^,^10^.

Then, the criterion for lethality is introduced as


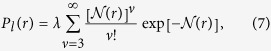


where *ν* is the number of simple lesions per cluster. The DSBs consequent to SSBs are more probable than those due to independent nearby SSB events^38^,^39^,^40^. This is accounted for by introducing the factor *λ* that is a probability of conversion of a SSB into DSB. The sum in Eq. (7) starts with *ν* = 3, which means that at least three simple damages (within a certain region) are required in order for damage to be lethal. The presence of coefficient *λ* requires that at least one of these lesions is converted to a DSB. This criterion, introduced in ref. 5 heuristically, is fully applicable for quantitatively correct prediction of cell survival, while the assumption that lethal damage is done either by a smaller (*ν* = 2) or by a larger (*ν* = 4) number of simple damages, yields systematically incorrect results (see Supplementary Fig. S1, SI). Function *P*_*l*_(*r*) represents the radial distribution of lethal lesions. Finally, it has to be integrated over the area perpendicular to the ion’s path and multiplied by the number density of sites on chromatin, *n*_s_, (assumed to be uniform) to obtain the number of lethal lesions per ion’s path d*x*:


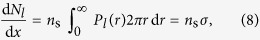


where 
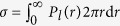
 is the effective cross section of the complex damage site, which depends on LET^5^. A detailed analysis of fluence of secondary electrons on a cylinder enwraping a DNA twist was performed in ref. 41 where the results of the analytical approach were compared to those of Monte Carlo simulations. Values of the parameters entering Eqs (6, 7, 8) as well as the references for more detailed explanation of these numbers are summarized in Supplementary Table S1 (SI). The number density of targets, *n*_s_, is proportional to the ratio of base pairs accommodated in the cell nucleus to the nuclear volume, *n*_s_~*N*_bp_/*V*_n_. The coefficient of proportionality takes into account that a target represents a double DNA twist comprising 20 bp^5^:


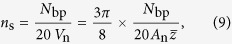


where the cross sectional area, *A*_n_, of the cell nucleus, its diameter, *D*_n_, and an average length of ions’ traverse through a nucleus, 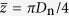, are listed in Table 1. Taking into account the chromatin dynamics during the cell cycle and that diploid cells contain a double set of chromosomes, one gets the final expression for *n*_s_:


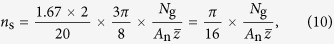


where *N*_g_ is genome size, equal to 3.2 Gbp for human cells^42^ and to 2.7 Gbp for Chinese hamster cells^43^. The factor 1.67 arises because of dependence of *N*_bp_ on the phase of the cell cycle. During interphase, the number density *n*_s_ remains constant during *G*_1_ phase, which takes about 1/3 of the total cell cycle duration (*T*_c_) in human cells^44^, but becomes doubled in the *S* and *G*_2_ phase after DNA replication has taken place. Averaging the number density of DNA over the different phases, one gets


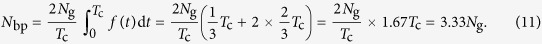


The obtained number densities of targets *n*_*s*_ for all cell lines considered in this study are listed in Table 1.

The probability Γ_e_ that a single electron hitting a DNA molecule induces a SSB, was taken to be equal to 0.03 in all the calculations. This value was estimated in ref. 5 by fitting the experimental probability for SSBs induced in plasmid DNA by secondary electrons as a function of dose^45^.

The effect of free radicals was also considered in ref. 5. Since the exact radial distribution of the probability of inducing a SSB by radicals is not known, it is assumed to be uniform within a certain distance from the ion’s path:


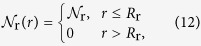


where *R*_r_ is the effective distance for free radicals propagation which depends on the projectile’s velocity and charge. In the presented analysis, we considered this value in the range between 5 and 10 nm. A uniform distribution of radicals within a certain distance from the ion’s path implies that the reactive species, formed in the nearest proximity to the path, are transported by a shock wave and their number density is nearly uniform inside the cylinder that enwraps the decayed shock wave^5^. The average probability 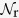 for SSBs caused by free radicals to take place was estimated as 0.08 from the comparison of the experimental results^45^ for plasmid DNA dissolved in pure water or in a scavenger-rich solution.

The value of 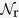 is also affected by environmental conditions of an irradiated target. In the case of hypoxic conditions, the value 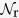 is reduced because the radical-induced damage may be repaired if oxygen is not present. The quantitatively correct description of the experimental survival probabilities of cells irradiated under hypoxic conditions was achieved by utilising the value 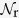 = 0.04 which is two times smaller than that in the aerobic environment; this corresponds to experimental data on the induction of DSBs and non-DSB clustered DNA lesions in mammalian cells at normal concentration of oxygen and at deep hypoxia^32^. Further work, however, is needed to explore, in more detail, how the probability of lethal lesion production by free radicals depends on the environmental conditions, e.g. at intermediate concentrations of oxygen^22^.

According to the analysis of ref. 5, the effect of each ion can be treated independently from others, since the average distance between the paths is considerably larger than the radii of tracks. Then, the number of lethal lesions per ion, traversing distance *z* through a cell nucleus is given by 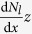 and the average number of lethal lesions per cell nucleus is given by^5^


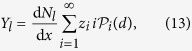


where the sum 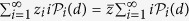 yields an average length of traverse of all ions passing through a cell nucleus for a given dose. The probability


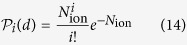


that exactly *i* ions traverse the cell nucleus depends on the average number of ions traversing it, *N*_ion_. The latter in its turn depends on dose, LET, and the size of cell nucleus: *N*_ion_ = *A*_n_*d*/*S*_e_, where *A*_n_ is the cross sectional area of the cell nucleus and *S*_e_ = |d*E*/d*x*| is a part of LET spent on ionization of tissue. At large values of *N*_ion_, 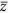 becomes dose-dependent. For values of *N*_ion_ relevant for this study, 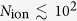, 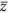 is nearly constant and substitution of Eq. (14) into (13) yields a linear dependence of the number of lethal lesions per cell nucleus on dose:


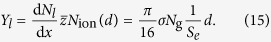


Equations (13) and (15) give the number of lethal damage sites per cell nucleus, therefore the probability Π_*l*_ of producing damage lethal to the cell is given by,


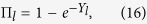


since a single lethal lesion is sufficient for the cell inactivation. Then, the probability of cell’s survival as a function of absorbed dose is given by unity less the above probability, see Eq. (2). When the probability of a successful repair of a complex lesion, *χ*, is introduced, Eq. (2) transforms into Eq. (5) which can be represented as


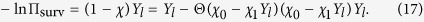


At *Y*_*l*_ < *χ*_0_/*χ*_1_, the survival probability in virtue of Eq. (15) is as follows,


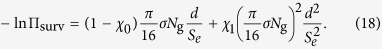


This result provides the molecular-level justification of the empirical LQ parameters *α* and *β* for doses 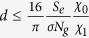:


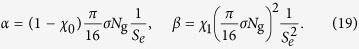


At *Y*_*l*_ > *χ*_0_/*χ*_1_, i.e. at 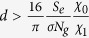, one derives the linear regime,


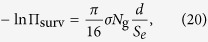


and the parameter α then transforms into


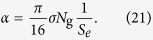


## Additional Information

**How to cite this article**: Verkhovtsev, A. *et al*. Multiscale approach predictions for biological outcomes in ion-beam cancer therapy. *Sci. Rep.* **6**, 27654; doi: 10.1038/srep27654 (2016).

## Supplementary Material

Supplementary Information

## Figures and Tables

**Table 1:** Characteristics of the cells studied.

cell line	*A*_n_(*μ*m^2^)	*D*_n_(*μ*m)	Ref.	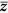 (*μ*m)	*n*_*s*_ (nm^−3^)
A549		9.6	46	7.5	1.2 × 10^−3^
AG1522	144 ± 45	13.4	23	10.6	4.2 × 10^−4^
HeLa	219 ± 3.5	16.7	47	13.0	2.2 × 10^−4^
NB1RGB	172 ± 2.2	14.8	48	11.6	3.1 × 10^−4^
A172	209 ± 3.2	16.3	49	12.7	2.4 × 10^−4^
EAhy926	95 ± 23	10.9	50	8.5	7.9 × 10^−4^
V79	88	10.6	20	8.2	7.2 × 10^−4^
CHO	127 ± 1.2	12.7	47	9.9	4.2 × 10^−4^

Cross sectional area, *A*_*n*_, and diameter, *D*_*n*_, of the cell nucleus, as well as an average length of ions’ traverse through a nucleus, 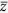, and the number density of complex damage sites on chromatin, *n*_*s*_, for different cell lines considered in this study. Experimentally measured values of *A*_*n*_ and *D*_*n*_ are taken from the indicated references.
