# Supplementary material for: Multiscale approach predictions for biological outcomes in ion-beam cancer therapy
Source: Sci Rep. 2016 Jun 14;6:27654. doi: 10.1038/srep27654 (PMC4906349; doi:10.1038/srep27654)
Supplement: Supplementary Information [file srep27654-s1.pdf]

Supplementary Information
"Multiscale approach predictions for biological outcomes in ion-beam cancer therapy"

Alexey Verkhovtsev,[1,2,*] Eugene Surdutovich,[3,1,†] and Andrey V. Solov'yov[1,‡]

[1]*MBN Research Center, Altenhöferallee 3, 60438 Frankfurt am Main, Germany*

[2]*Instituto de Física Fundamental, CSIC, Serrano 113-bis, 28006 Madrid, Spain*

[3]*Department of Physics, Oakland University, Rochester, Michigan 48309, USA*

---

[*] verkhovtsev@iff.csic.es

[†] surdutov@oakland.edu

[‡] solovyov@mbnresearch.com; On leave from A.F. Ioffe Physical-Technical Institute, 194021 St. Petersburg, Russia

TABLE S1. **Values of parameters used in calculations.**

Parameter	Value	Reference (in Ref. 1)
$\mathcal{F}_{\mathrm{e}}(r)$		Eq. (20)
Γ_{e}	0.03	Sect. 4.3.1
$\mathcal{N}_{\mathrm{r}}$ (normal)	0.08	Sect. 4.4
$\mathcal{N}_{\mathrm{r}}$ (hypoxic)	0.04	this work
λ	0.15	Sect. 4.3.1
$P_l(r)$		Sect. 6.1 and Fig. 13

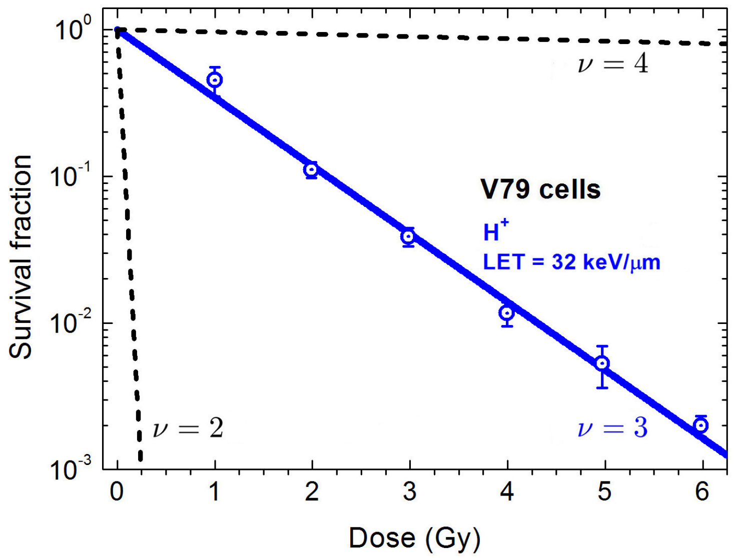

FIG. S1. **Dependence of cell survival after irradiation with ions on the criterion for lethality of radiation damage.** All the calculations performed in this work were carried out assuming that at least three simple damages within two DNA twists are required in order for damage to be lethal (the sum in Eq. (7) in the *Methods* section starts with $\nu = 3$). The variation of this criterion, i.e. when it is supposed that lethal damage is done by at least two ($\nu = 2$) or by four ($\nu = 4$) simple damages, leads to systematically incorrect survival curves (dashed lines). Symbols denote the experimental data for Chinese hamster V79 cells [2].

[1] Surdutovich, E. & Solov'yov, A. V. Multiscale approach to the physics of radiation damage with ions. *Eur. Phys. J. D* **68**, 353 (2014).

[2] Folkard, M. *et al.* The irradiation of V79 mammalian cells by protons with energies below 2 MeV. Part I: experimental arrangement and measurements of cell survival. *Int. J. Radiat. Biol.* **56**, 221–237 (1989).
